# Scoliosis and sagittal balance in Parkinson’s Disease: analysis of correlations

**DOI:** 10.1186/1748-7161-9-S1-O38

**Published:** 2014-12-04

**Authors:** Luciano Bissolotti, Sabrina Donzelli, Massimiliano Gobbo, Fabio Zaina, Jorge Hugo Villafane, Stefano Negrini

**Affiliations:** 1 Rehabilitation Service, Fondazione Teresa Camplani Casa di Cura Domus Salutis, Brescia, Italy; 2 ISICO, Milan, Italy; 3 Department of Clinical and Experimental Sciences, University of Brescia, Brescia, Italy; 4 IRCCS Fondazione Don Gnocchi ONLUS, Milan, Italy; 5 IRCCS Fondazione Don Gnocchi ONLUS, Milan, Italy, ISICO, Milan, Italy

## Background information

The knowledge concerning scoliosis in Parkinson’s Disease (PD) and its correlations with sagittal balance (SB) is sparse.

## Purpose

The aim of this study was to describe the prevalence of scoliosis in PD patients and the existing correlations with SB in relation to the spinopelvic morphology.

## Methods

64 consecutive PD patients were included: 53 males, 11 females; 69.5±8.1 years; 5.6±4.1 years of disease (YOD); Hoehn Yahr (H&Y) 2.4±1.1, UPDRS-M 16.1±12.5. The clinical assessment included HY and UPDRS-M score, Pain NRS 0-10 and trunk rotation in bending (ATR). Lumbar lordosis (LL), thoracic kyphosis (TK), scoliosis curves (SC), spinosacral angle (SSA), spinopelvic angle (SPA), pelvic incidence (PI), sacral slope (SS) and pelvic tilt (PT) were radiographically assessed. Patients have been compared according to the presence of SC >10° (PDts) Cobb or the absence of SC (PDns).

## Results

45% of cohort presented a SC larger than 11°, 84% of the patients in PDts presented a thoracolumbar curve, 10% a thoracic one and 6% a lumbar one. They did not present differences with PDns about age (70.8±6.8 vs 68.8±8.6yrs) and YOD (6.6±6.0 vs 6.1±4.3yrs). No differences have been detected for H&Y score (2.6±1.2 vs 2.6±1.1) and UPDRS-M (16.5±12.3 vs 16.8±12.4). Pain was slightly higher in PDts than PDns (3.2±2.7 vs 2.3±2.8). ATR was higher in PDts (5.0±4.3 vs 1.5±2.0, p<0.01). TK (47.1±14.5 vs 45.5±12.5°), LL (47.5±24.2 vs 50.3±13.9°), SSA (111.7±22.3 vs 120.1±10.9°) and SPA (154.4±18.2 vs 156.9±12.3°) were not different (p>0.05). PI (55.3±11.2 vs 53.6±11.4°) and PT (21.6±13.2 vs 16.8±8.1°) were slightly but not statistically different, while SS was not (34.7±11.2 vs 36.2±9.3°).

## Conclusions and discussion

Pelvic parameters did not differ significantly in the two groups. The prevalence of scoliosis in PD was higher than previously described by the other Authors and thoracolumbar spine was the mostly affected. SB was not different between two groups while, in PDts, spinopelvic parameters presented the tendency to have a larger PT. Further studies will deepen this issues, in PD patients with more severe scoliosis.

